# Effect of heat‐moisture treatment on the structural and physicochemical characteristics of sand rice (*Agriophyllum squarrosum*) starch

**DOI:** 10.1002/fsn3.2622

**Published:** 2021-10-20

**Authors:** Chunsen Wu, Guiying Ji, Fan Gao, Jian‐Ya Qian, Liang Zhang, Qian Li, Chen Zhang

**Affiliations:** ^1^ School of Food Science & Engineering Yangzhou University Yangzhou China; ^2^ China‐Canada Joint Lab of Food Nutrition and Health (Beijing) Beijing Technology and Business University Beijing China

**Keywords:** digestibility, heat‐moisture treatment, sand rice starch, structure

## Abstract

A small granule starch from sand rice (*Agriophyllum squarrosum*) was subjected to heat‐moisture treatment (HMT) at different moisture contents (MCs,15%–30%). With MC≤20%, a higher MC resulted in increases in the starch orders (i.e., short‐range and crystalline structure) with unchanged granule morphology. Nonetheless, a further elevated MC (>20%) gradually destroyed the granule morphology and starch orders. Also, HMT gradually vanished the lamellar structure as MC increased during HMT. These structural evolutions in HMT‐modified starch resulted in greater thermal stability, higher pasting temperature, lower pasting viscosity and weakened digestibility. Particularly, HMT applied directly in sand rice starch at 20% MC obtained the highest amount of SDS and RS (23.6%), which was 2.2‐fold higher than that of native starch. Therefore, the small granule sand rice starch can be modulated by HMT through controlled MC to expand their application range in food production.

## Graphical abstract

A small granule starch was modified by HMT. A higher MC (≤20%) during HMT increased starch orders with unaltered granule morphology, and HMT‐20% produced the highest amount of SDS and RS in starch. The sand rice starch can be modulated by HMT through controlled MC.

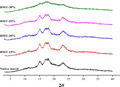

## 1 INTRODUCTION

Sand rice (*Agriophyllum squarrosum*) is an extensively cultivated psammophyte in the desert area of Asia for its sand stabilization ability (Qian et al., 2016; Zhao et al., 2014). For local people, sand rice has been popularly consumed as the comestible and medicine for hundreds of years (Xu et al., 2018). As reported, the nutritional value of sand rice is deemed to be comparable to that of quinoa (*Chenopodium quinoa* Willd.), who has gained global recognition with unique nutritional characteristics and adaptability (Zhang et al., 2018). Thus, the sand rice will be a potential crop as the alternative food for growing populations.

Increasing demand for more sand rice production requires further understanding and processing them. In sand rice, starch is the most abundant carbohydrate making up 32.5%–51.2% (Peng et al., 2018). This starch is reported as an A‐type starch with round shape smaller than 2 μm in diameter, which has a similar size to the Amaranth starch (Bet et al., 2018; Peng et al., 2018). In practice, the amaranth starch has been used as a fat substitute because of its size similar to the lipid micelles (Bet et al., 2018). It is believed that this small granule starch from sand rice is a promising candidate as the ingredient in food industry.

Currently, starch in its native form is restricted in application, and several processing methods are employed to improve its properties and uses (Wang et al., 2020). A primary and safe modification of starch for food application is through hydrothermal modification (Asare et al., 2021; Kaur et al., 2019; Nie et al., 2019; Zhao et al., 2021). Herein, heat‐moisture treatment (HMT) is a physical interaction‐reforcing technique that improves the functional and physical properties of starch with no alteration to the original molecular composition (Wang et al., 2020). It has been widely acknowledged that HMT modification is controlled by the botanical sources, moisture level, heating time, and temperature (Zhao et al., 2021). Numerous studies have been conducted on HMT‐modified normal starches, such as corn, wheat, potato, rice, buckwheat, and tapioca starch (Hoover, 2010). However, scant information is acquired on the sand rice starch treated by HMT. Based on these, the current work was aimed to investigate changes of the structure and physicochemical properties of sand rice starch after HMT exertion. We believe our results will pave the way for future use of this small granule starch from sand rice in the food industry.

## 2 MATERIALS AND METHODS

### 2.1 Materials

Sand rice (*Agriophyllum squarrosum*) was harvested in the Gansu province of China. The *ɑ*‐amylase from the hog pancreas (50 U/mg) was obtained from Sigma‐Aldrich (China) Co., Ltd. The amyloglucosidase (200 U/ml) was purchased from Megazyme International Ireland Ltd. Glucose oxidase‐peroxidase assay kit was bought from the Applygen Technologies Inc. All other chemicals of analytical grade were obtained from Sinopharm Chemical Reagent Co., Ltd.

### 2.2 Starch isolation

The starch was isolated using the alkaline method with minor modification (Goel et al., 2020). Sand rice was dehulled and steeped in 0.02% NaOH solution at 4ºC for 12 h. The mixture was mashed and filtered with a 100‐mesh sieve, then centrifuged at 3,000 × *g* for 10 min. After then, the supernatant and protein layer were discarded. The procedure was repeated several times until no protein observed. The precipitate was dried at 40ºC for 48 h, and passed through a 100‐mesh sieve to obtain the sand rice starch.

### 2.3 HMT

HMT modification was done with a method described by Liu et al. with some modifications (Liu, Guo, et al., 2015). The moisture content (MC) of sand rice starch was adjusted to desired value (15%, 20%, 25%, and 30%) using deionized water, then equilibrated at 4℃ for 24 h. After then, 30 g (dry basis) of each starch sample with adjusted MC was sealed and heated at 110℃ for 8 h. Afterward, these starches were dried at 40℃ for 24 h, grounded into powder using a mortar and sifted through a 100‐mesh sieve.

### 2.4 Scanning electron microscopy (*SEM*)

An environmental scanning electron microscope (GeminiSEM 300, Carl Zeiss, Germany) was applied to evaluate the granule morphology at an accelerating voltage of 10.0 kV. Before observation, every starch sample was spread onto circular metal stubs and sputtered with gold in a sputter coater (BAL‐TEC SCD 500, Leica, Liechtenstein).

### 2.5 Attenuated total reflection‐Fourier transform infrared spectroscopy (ATR‐FTIR)

The ATR‐FTIR analysis was conducted using an IS10 FTIR spectrometer (Thermo Electron Corporation) assembled with an attenuated total reflection accessory and a deuterated triglycine sulphate. Each sample was scanned for 32 times from 1,200 to 800 cm^−1^ with a resolution of 4 cm^−1^. The spectrum was smoothed, baseline corrected and deconvoluted by the OMNIC software (Thermo Scientific). The peak intensity at the wavenumber of 1,047 cm^−1^ was divided by that at 1,022 cm^−1^ to calculate the intensity ratio R_1047/1022_.

### 2.6 X‐ray diffraction (XRD)

The powder method of XRD was applied to starch samples using a Bruker D8‐advance X‐ray diffractometer (Bruker AXS Inc.) (Wu et al., 2017). XRD pattern was scanned in a range from 3° to 35° (2*θ*) at 40 kV and 4 mA, with a step size of 3° per min. The relative crystallinity was quantitatively estimated by comparing area under the crystalline peaks with the total area of the crystalline and amorphous regions.

### 2.7 Small‐angle X‐ray scattering (SAXS)

SAXS system (NanoSTAR, Bruker AXS, Karlsruhe, Germany) was operated at 0.6 mA and 50 kV, using Cu‐Kα radiation with a wavelength (λ) of 0.154 nm as X‐ray source. The determination of starch samples (ca. 60% MC) were made between 0.2° and 3.2° for 15 min, and further processed with DIFFRAC^plus^ NanoFit software. A relationship between scattering vector (*q*) and scattering angle (*θ*) was calculated according to a formula q=4πsinθ/λ (Suzuki et al., 1997).

### 2.8 Differential scanning calorimeter (DSC)

The thermal properties of starch samples were investigated with a DSC 8,500 Thermal Analytical System (PerkinElmer, USA). The distilled water (6 μl) was added into starch (3.0 mg) in an aluminum pan. This mixture was then immediately sealed and equilibrated at 4℃ for 24 h. Each sealed sample was heated form 20℃ to 120℃ at a rate of 10℃/min, taking the onset temperature (T_o_), peak temperature (T_p_), conclusion temperature (T_c_), gelatinization enthalpy (ΔH), and temperature range (T_c_‐T_o_).

### 2.9 Rapid visco‐analysis (RVA)

Pasting properties were analyzed by a rapid viscosity analyzer (RVA4500, Perten Instruments Ltd.) with paddle rotating 160 rpm at a starch concentration of 6% (w/w, dry basis). The starch slurry was firstly held at 50℃ for 1 min, heated from 30℃ to 95℃ at a rate of 5.4 ℃/min, held at 95℃ for 5 min, cooled from 95℃ to 30℃ at the same rate, and then maintained at 50℃ for 1 min. Recorded attributes were the peak viscosity (PV), breakdown (BD), final viscosity (FV), setback (SB), and peak temperature (PT).

### 2.10 In vitro digestibility

The digestible features of starches were evaluated according to the partially modified method of *Englyst* (Englyst et al., 1992). The starch sample (0.5 g, dry basis) in 15 ml of sodium acetate buffer (0.2 mol/L, pH 5.2) was stirred in a boiling water bath (100℃) for 20 min. Then, the sample was incubated in a 37℃ water bath with agitation (50 rpm). Afterward, 10 ml of blended enzymes containing 3,000 U of α‐amylase and 200 U of amyloglucosidase was added in each sample. Then, a glucose test kit (GOPOD kit) was used to determine the amount of glucose produced exactly at 20 min (G20) and 120 min (G120), together with the free glucose (FG) in the native starch. The rapidly digestible starch (RDS) is a starch fraction that is digested in 20 min, slowly digestible starch (SDS) is a starch fraction that is digested between 20 and 120 min, and resistant starch (RS) is an undigested fraction within 120 min.

### 2.11 Statistical analysis

Assays were performed in triplicate for each sample and the results were expressed as the mean value ± standard deviations. Data analyses and charts plotting were conducted using SPSS 18.0 and Origin 8.5 with Duncan's test for significant difference declared at *p* ≤ .05.

## 3 RESULTS AND DISCUSSION

### 3.1 Granule morphology

**FIGURE 1:**
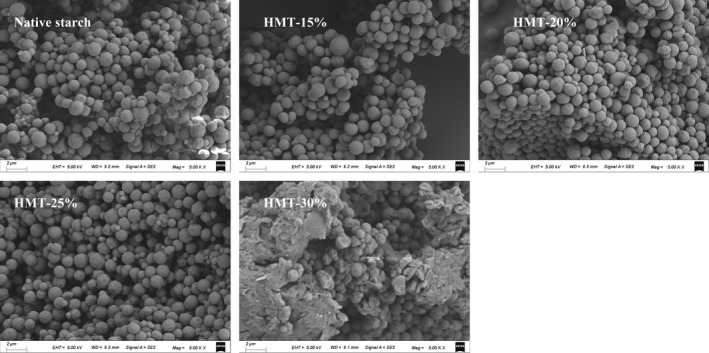
*SEM* images of the native and HMT‐modified sand rice starches. (15%, 20%, 25%, or 30% represents the treated moisture content)

The *SEM* micrographs of native and HMT‐modified sand rice starch are presented in Figure 1. The native starch granules had spherical shape with diameters mostly less than 1 μm, which is similar to the granule size of amaranth starch (Bet et al., 2018). The small size of its granules suggests that there will be various applications for sand rice starch such as fat substitute and thickener in sauces. Besides, there was no significant difference in granule morphology between native starch and its HMT‐modified counterparts at low moisture levels (e.g., 15% and 20%). It was obviously that with a limited MC (≤20%), limited hydration appeared to hardly alter the granule morphology of starch. Further elevated MC at 25% led to a coarser granule surface. When the MC of 30% was used, most granules were disrupted with agglomerates existing. These results showed that HMT at a high MC could destroy the granule morphology because of the partial gelatinization of starch (Liu et al., 2015).

### 3.2 ATR‐FTIR

**FIGURE 2:**
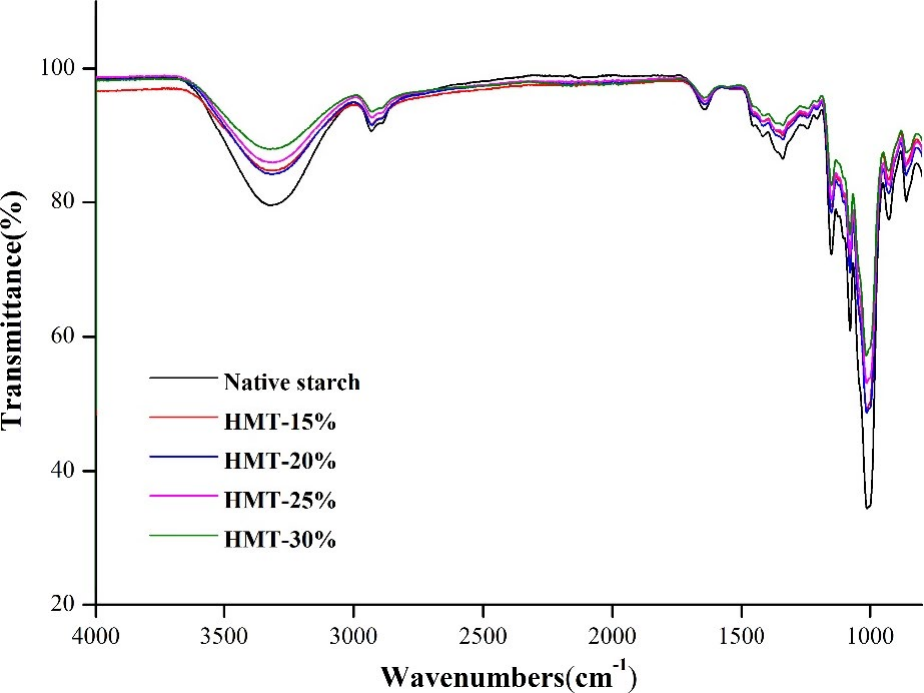
ATR‐FTIR spectra for the native and HMT‐treated sand rice starches. (15%, 20%, 25%, or 30% represents the treated moisture content)

The ATR‐FTIR spectra of native and HMT‐modified starches are displayed in Figure 2. The absorption peaks in the spectra were not moved after HMT exertion, indicating that HMT led to physical modification without the emergence and disappearance of certain functional groups. The absorption peak ranged of 3,100–3,700 cm^−1^ was gradually lowered by increasing MC, representing that HMT might generate the interaction of hydrogen bonds between molecules. The bands at 800–1,200 cm^−1^ are recognized as the fingerprints of starch, which reflect the stretching vibration of starch C‐H,C‐OH and C‐C, determining the starch conformation and hydration process (Chen et al., 2012). The absorption intensity (800–1,200 cm^−1^) of HMT‐modified starch was lowered than in native starch due to the firm conformation modification. The bands at 1,047 and 1,022 cm^−1^ are associated with ordered/crystalline and amorphous regions in the external region of the starch granule (Man et al., 2014). The ratio (*R*_1047/1022_) of intensity at 1,047 cm^−1^ to that at 1,022 cm^−1^ was applied to quantify the degree of order in starch (Liu et al., 2019; Soest et al., 1995). In Table 1, R1047_/1022_ values increased to 0.658 with the treating MC rose to 20%, and then gradually decreased with further elevated MC. This changing trend is similar to the HMT‐treated highland barley starch (Liu et al., 2019). These results suggested that the starch chains rearranged during HMT at low MC (≤20%); but short‐range ordered structure could be disrupted certainly at a higher MC (30%).

**TABLE 1:** R_1047/1022_, XRD, and SAXS characteristics of native and HMT‐modified sand rice starches

Samples	R_1047/1022_	Relative crystallinity (%)	SAXS		
			q(nm^−1^)	d (nm)	PI (a.u.)
Native starch	0.636 ± 0.005^c^	22.0 ± 0.5^b^	0.67 ± 0.00^a^	9.37 ± 0.00^a^	81.2 ± 0.64^a^
HMT−15%	0.643 ± 0.004^b^	23.6 ± 0.3^ab^	0.67 ± 0.01^a^	9.37 ± 0.17^a^	66.4 ± 0.96^b^
HMT−20%	0.658 ± 0.002^a^	24.2 ± 0.2^a^	0.67 ± 0.00^a^	9.37 ± 0.00^a^	33.2 ± 0.78^c^
HMT−25%	0.649 ± 0.006^b^	20.7 ± 0.1^c^	0.67 ± 0.01^a^	9.37 ± 0.17^a^	6.98 ± 0.38^d^
HMT−30%	0.621 ± 0.007^d^	10.1 ± 0.2^d^	0.65 ± 0.01^a^	9.65 ± 0.17 ^a^	1.31 ± 0.23^e^

Note

R_1047/1022_ is the ratio of relative intensity at peak 1,047 cm^−1^ to that at 1,022 cm^−1^. Parameters obtained by SAXS: q, peak position of semicrystalline lamellae; d, the average repeat distance of semicrystalline lamellae; PI, the scattering peak intensity.

### 3.3 XRD

**FIGURE 3:**
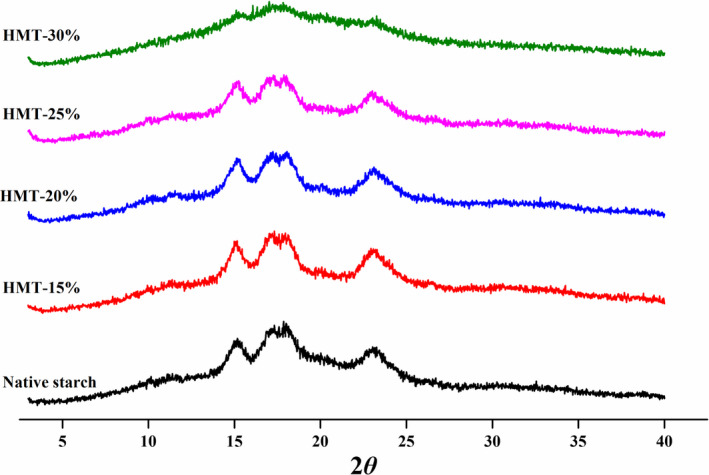
X‐ray diffraction pattern of the native and HMT‐modified sand rice starches. (15%, 20%, 25%, or 30% represents the treated moisture content)

The XRD patterns of native and HMT‐modified sand rice starch are showed in Figure 3. The X‐ray diffraction peaks appeared at 2*θ* (15.1° and 23.1°), and a double peak at 2*θ* (17.2° and 18.0°), pointing out that sand rice starch is an A‐type starch. The HMT‐modified starches still had the similar XRD patterns, indicating that the A‐type crystalline form was retained when exposed to HMT. Previously studies have demonstrated that the crystalline type of buckwheat, cassava and sorghum starch could hardly be changed through HMT (Gunaratne & Hoover, 2002; Sun et al., 2014; Xiao et al., 2017). The relative crystallinity (seen in Table 1) for native starch is 22.0%, and increased from 23.6% to 24.2% as MC increased from 15% to 20%, then gradually reduced with further increased MC. In particular, HMT‐30% showed the greatest reduction in the relative crystallinity. As reported in literature, excessive moisture during HMT could sharply reduce the crystallinity of breadfruit starch(Tan et al., 2017). These results suggested that low MC (≤20%) could improve the crystalline structures of sand rice by promoting starch chain reassembly, then modification at higher MC (30%) resulted in disruption of starch crystallites.

### 3.4 Lamellar structure

**FIGURE 4:**
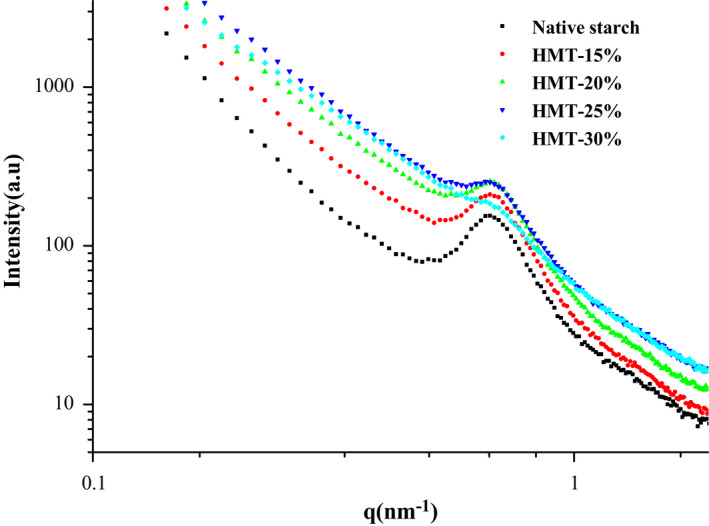
Double logarithmic SAXS patterns of the native and HMT‐modified sand rice starches. (15%, 20%, 25%, or 30% represents the treated moisture content)

The information about lamellar structure of alternating crystalline and amorphous regions with a regular distance of 9–10 nm can be characterized by SAXS (Zhang et al., 2014). In Figure 4, the double logarithmic SAXS patterns of native and HMT‐modified sand rice starch are displayed. The scattering intensity (I) as vertical ordinate is in direct proportion to the scattering vector as abscissa. A scattering peak at around q = 0.67 nm^−1^ was observed, which is related to the semi‐crystalline region of the starch granule (Tan et al., 2015). According to the law of Bragg, the thickness values of semi‐crystalline lamella calculated form d = 2π/q are listed in Table 1. As can be seen, HMT does not significantly influence the location of the scattering peak.

The value of peak intensity hinges on the degree of the semi‐crystalline structure, the lower the peak intensity, the less compact semi‐crystalline regions of starch (Wu et al., 2019). As seen in Figure 4 and Table 1, the peak intensity at ca. 0.67 nm^−1^ was gradually reduced as the moisture level increased, and it almost disappeared for HMT‐30. A more sharp decreasing trend can be seen at a higher MC during HMT. These results indicate that the original semi‐crystalline lamellar structure was gradually damaged as the MC increased during HMT to a certain degree.

### 3.5 Thermal properties

The values obtained for the transition temperatures and gelatinization enthalpy (△*H*) of the native and HMT‐modified starches are presented in Table 2. The *T*_o_, *T*_p_, *T*_c_ and *T*_c_‐*T*_o_ of starch increased after HMT, while the △*H* significantly decreased, and all these changes were correlated well with the MC during HMT. These results are in agreement with the previous studies on buckwheat starch and rice starch (Khunae et al., 2007; Liu, Guo, et al., 2015). The melting temperature (*T*_o_, *T*_p_, and *T*_c_) of crystallites are controlled indirectly by the surrounding amorphous region in the starch granule (Gunaratne & Hoover, 2002). HMT with a higher MC may led to more closely packed in the crystalline and amorphous region, then reduce the destabilization effect of the amorphous region on the crystallite melting (Tan et al., 2017). The increased *T*_c_‐*T*_o_ values with a higher MC might be induced by the reorganized of starch chains in promoting the formation of some ordered and stable structure. The gradual reduction of △*H* with increased MC was related to the increased disruption of the amorphous region and association of double helices within starch granules during HMT (Tan et al., 2017).

**TABLE 2:** Thermal properties of native and HMT‐modified sand rice starches

Samples	*T*_o_(ºC)	*T*_p_(ºC)	*T*_c_(ºC)	*T*_c_‐ *T*_o_ (ºC)	△H (J/g)
Native starch	70.9 ± 0.2^c^	75.1 ± 0.4^c^	79.3 ± 0.5^e^	8.4 ± 0.5^d^	10.9 ± 0.4^ab^
HMT−15%	71.5 ± 0.5^c^	76.2 ± 0.7^bc^	81.4 ± 0.6^d^	9.9 ± 0.6^c^	11.3 ± 0.5^a^
HMT−20%	72.5 ± 0.8^bc^	76.6 ± 0.4^b^	83.1 ± 0.7^c^	10.6 ± 0.5^bc^	9.7 ± 0.6^b^
HMT−25%	73.8 ± 0.7^b^	79.1 ± 0.3^a^	85.3 ± 0.8^b^	11.5 ± 0.7^b^	7.6 ± 0.4^c^
HMT−30%	75.7 ± 0.5^a^	78.9 ± 0.6^a^	89.1 ± 0.7^a^	13.4 ± 0.4^a^	2.8 ± 0.2^d^

Note

The different superscript letters declares different significantly (*p* < .05) in the same column.

Abbreviations: △H, gelatinization enthalpy;*T*_c_, completion temperature; *T*_o_, onset temperature; *T*_p_, peak temperature.

### 3.6 Pasting properties

The pasting parameters of native and HMT‐treated sand rice starches are listed in Table 3. The HMT‐modified starch had a higher PT than the native starch, and HMT‐25% displayed the highest PT of 82.3ºC. These results indicated that the thermostability was pronouncedly improved after HMT, which was meeting with the DSC results. As reported in the literature, an increase in pasting temperature could be attributed to the closely packed structures and limited swelling of starch granules after HMT (Liu et al., 2019; Xiao et al., 2017).

**TABLE 3:** Pasting properties and digestion features of native and HMT‐treated sand rice starches

Samples	PT(ºC)	PV (cp)	BD (cp)	FV (cp)	SB (cp)	RDS (%)	SDS (%)	RS(%)
Native starch	75.5 ± 0.2^e^	2,458 ± 62^a^	885 ± 31^a^	3,864 ± 86^a^	2,853 ± 49^c^	89.3 ± 0.5^a^	4.1 ± 0.2^c^	6.6 ± 0.2^c^
HMT−15%	78.4 ± 0.1^c^	2,271 ± 53^a^	234 ± 12^d^	3,083 ± 73^b^	2046 ± 37^b^	81.6 ± 0.7^b^	8.3 ± 0.4^b^	10.1 ± 0.3^b^
HMT−20%	79.5 ± 0.3^b^	1,001 ± 19^b^	518 ± 21^b^	2,952 ± 89^c^	1825 ± 22^a^	76.4 ± 0.9^d^	10.2 ± 0.5^a^	13.4 ± 0.6^a^
HMT−25%	82.3 ± 0.4^a^	424 ± 11^c^	318 ± 13^c^	2,934 ± 33^c^	1,218 ± 19^d^	79.1 ± 0.8^c^	10.7 ± 0.3^a^	10.2 ± 0.5^b^
HMT−30%	76.7 ± 0.2^d^	283 ± 8^d^	154 ± 11^e^	1,253 ± 17^d^	824 ± 11^e^	82.2 ± 0.6^b^	8.5 ± 0.5^b^	9.3 ± 0.4^b^

Note

The different superscript letters in a column declares different significantly (*p* < .05) in the same column.

Abbreviations: BD, breakdown viscosity; FV, final viscosity; PT, pasting temperature; PV, peak viscosity; SB, setback viscosity.

The PV, BD, FV, and SB of all the HMT‐modified sand rice starch were significantly lower than those of the native counterparts. The highest viscosity in native sand rice starch was partially attributed to a complete rupture of starch granules during pasting. And the higher SB was due to that the cooling of pasting was rendered easier as the highly dispersed amylose (Watcharatewinkul et al., 2009). The decrement in BD represented that HMT‐modified starches were stable upon continues heating and shearing, which was in accordance with the higher PT of all modified samples. Liu et al. reported that the decreased BD was due to noticeably lower amounts of amylose leached from the restricted and rigid swollen granules in the HMT‐modified starches (Liu et al., 2015).

The higher PT and lower paste viscosity implied that the sand rice starch granules were strengthened by HMT. There might be two reasons for this: firstly, the short‐range ordered, crystalline and lamellar structure were rearranged during HMT with enhanced thermal stability; and secondly, there was a relatively compact structure in HMT‐modified samples with the enhancement of intra‐molecular bonding requiring more energy for pasting (Adebowale et al., 2005; Tan et al., 2017).

### 3.7 In vitro digestibility

The in vitro digestion results of native and HMT‐treated sand rice starches are presented in Table 3. The RDS, SDS, and RS of native sand rice starch were 89.3%, 4.1%, and 6.6%, respectively. It can be seen that HMT decreased RDS content but increased SDS and RS contents. Especially, when the MC was 20%, the total amount of SDS and RS substantially increased to 23.6%, which was 2.2‐fold higher than the native starch. Meanwhile, HMT‐20% had the highest RS content (13.4%). These results revealed that the moisture during HMT was crucial adjective in modulating the starch digestibility.

For sand rice starch, it is suggested that HMT induced rearrangement/disruption within the starch structure, thus modulating the pasting and digestion properties. The modification effect strongly depends on the moisture level during HMT. With a moderate MC (≤20%), simultaneous increases in short‐range and long‐range orders with slightly modified morphology can be seen. Nonetheless, when a higher MC (≥25%) was used, HMT might promote the starch structure disruption rather than rearrangement (shown by significantly decreased order structures). Also, HMT gradually damaged the starch lamellar structure as the moisture level increased. These structural evolutions were positively related to the transformation of RDS into SDS/RS upon HMT modification. It was proposed that the newly formed starch orders had relatively high perfection with less flaws, higher stability and greater resistance to enzyme hydrolysis (Liu et al., 2019).

## 4 CONCLUSION

To conclude, HMT had a marked impact on the structural and physicochemical attributes of sand rice starch. The increased MC (up to 20%) improved the molecular order structure (i.e., short‐range and crystalline structure) but unchanged the granule morphology; further elevated MC over 20% led to a gradual disruption of starch orders and granule structure. Meanwhile, HMT gradually vanished the lamellar structure with the elevated MC during HMT. Compared with native starch, HMT‐modified starch had higher pasting temperature, lower paste viscosity and weakened digestibility. Particularly, HMT‐20% gained the highest amount of SDS and RS at 23.6%. Generally, MC played a crucial role in controlling the structure and properties of the HMT‐modified sand rice starch for practical application. These results here help us in well understanding the effects of HMT modification from the structural view, which thus are beneficial for the production of sand rice starch products with tailored digestibility and pasting properties.

## CONFLICT OF INTEREST

The authors have no conflict of interest to declare.

## Data Availability

All the data alongside our manuscript is available.
